# Editorial: Reviews in neuropharmacology 2023: microbiota gut-brain axis, therapeutic insights for neurodegenerative diseases

**DOI:** 10.3389/fphar.2024.1499072

**Published:** 2024-10-03

**Authors:** Bhagavathi Sundaram Sivamaruthi, Natarajan Suganthy, Carolina Pellegrini

**Affiliations:** ^1^ Office of Research Administration, Chiang Mai University, Chiang Mai, Thailand; ^2^ Innovation Center for Holistic Health, Nutraceuticals, and Cosmeceuticals, Faculty of Pharmacy, Chiang Mai University, Chiang Mai, Thailand; ^3^ Bionanomaterials Research Lab, Department of Nanoscience and Technology, Alagappa University, Karaikudi, India; ^4^ Department of Clinical and Experimental Medicine, University of Pisa, Pisa, Italy

**Keywords:** gut-brain axis, microbiota, neurodegenerative diseases, probiotics, Alzheimer’s disease, Parkinson’s disease

Neurodegenerative diseases (NDs), such as Alzheimer’s and Parkinson’s disease (PD), represent a major healthcare challenge due to their significant socioeconomic impact worldwide (Toader et al., 2023). Mounting evidence for the involvement of the gut-brain axis (GBA) in the pathogenesis and progression of these NDs opens up a new frontier in research. The gut microbiota plays a critical role in modulating the GBA, and the potential therapeutic implications of this understanding are vast, inspiring us to explore and develop this area further (Thangaleela et al., 2022).

The Research Topic, “*Reviews in Neuropharmacology 2023: Microbiota Gut-Brain Axis, Therapeutic Insights for Neurodegenerative Diseases*,” collected five reviews (two systemic reviews, two reviews and one mini-review) in different aspects of the field.

The systematic review by Ankul et al. intended to report how monosodium glutamate (MSG) affects the brain and causes NDs. The study collected relevant literature from several scientific databases and selected specific studies for the discussion. This review details the link between glutamate toxicity and Alzheimer’s disease (AD). The review claimed that the increased level of glutamine and glutamate in the brain is associated with MSG dose. Ultimately, MSG is closely related to memory impairment, neuronal atrophy, and AD-like pathology. The study stated that AD-like pathology, like neuronal shrinkage and memory impairments, was shown by MSG at an early age. The consumption of MSG during pregnancy may affect the cognitive development of offspring.

Another systematic review of the Global research status and trends of enteric glia deals with the field as a bibliometric analysis. Papers such as these help to determine the research highlights, global status, and future directions of the specific field. Li et al. reviewed about 514 research papers from 36 countries. The United States of America was distinguished as the most influential country in enteric glia research. The University of Nantes and Michigan State University placed in the top rank. It has been identified that Crohn's disease was a subject of debate on enteric glia, and inflammation, gut microbiota, and intestinal mobility might be the interest of research.

Gut microbiota plays a critical role in NDs, and its products can affect brain function via GBA. The microbiota of PD subjects differs from that of non-PD subjects, and the composition variation influences the microbial metabolites. Especially the one of the short-chain fatty acids (SCFAs), butyrate, plays an important role in regulating the host inflammatory responses. In this Research Topic, Elford et al. discussed the changes in the gut microbial composition and butyrate with special reference to PD pathology. The authors claimed that butyrate’s role is complex, and further studies are needed to explain whether the butyrate concentration is associated with the onset of PD and its severity.

Similarly, Zhang et al. discussed the impact of gut microbial metabolites as therapeutic targets for AD. The authors detail the bidirectional communication of the gut and brain and the regulatory effects of metabolites on AD pathogenesis and therapy by modulating the gut microbial niches. The study highlights the intestinal barrier’s structure and defensive functions and discusses microbial products, their producers, and their effects on AD. The study features the functions of microbial metabolites, including SCFAs neurotransmitters, and their mechanisms behind AD pathology. Possible therapeutic strategies, like lifestyle changes, probiotics, prebiotics, and antibiotics, have also been discussed targeting gut microbiota modulation for AD.

Kumar et al. mini-review discussed the role of probiotics and the GBA in cognitive development. Probiotics are potent enough to regulate the gut microbiota positively. Several pre-clinical and clinical trials suggested that probiotic interventions improved the mild cognitive impairment and anti-inflammatory system of the host. The study suggested that the beneficial effects of probiotics are possibly due to their impact on gut microbiota. The report suggested that further detailed studies on this domain are required to demonstrate the therapeutic potential of probiotics to manage cognitive declines.

In conclusion, the review articles published on the Research Topic help us understand the immense role of gut microbiota and their metabolites in preventing and managing NDs. The articles also explain how food ingredients, like MSG, are related to early-stage AD-like behaviors. An article highlights the global distribution and research domain of enteric glia research. Together, it has been clear that further studies are needed to deepen our understanding of microbiota, GBA, and their therapeutic insights to manage NDs.

## References

[B1] Thangaleela S. Sivamaruthi B. S. Kesika P. Bharathi M. Chaiyasut C. (2022). Role of the gut–brain Axis, gut microbial composition, Diet, and probiotic intervention in Parkinson’s disease. Microorganisms 10 (8), 1544. 10.3390/microorganisms10081544 36013962 PMC9412530

[B2] Toader C. Dobrin N. Brehar F. M. Popa C. Covache-Busuioc R. A. Glavan L. A. (2023). From recontion to remery: the significance of biomarkers in neurodegenerative disease pathology. Int. J. Mol. Sci. 24 (22), 16119. 10.3390/ijms242216119 38003309 PMC10671641

